# The COVID-19 pandemic: besides “post-truth” and “post-capitalism”, should we also consider “post-education” and “post-reason”?

**DOI:** 10.7189/jogh.11.01003

**Published:** 2021-04-03

**Authors:** Igor Rudan

**Affiliations:** Centre for Global Health, The Usher Institute, The University of Edinburgh, Scotland, UK

With more than 100 000 scientific papers having been published about the new coronavirus throughout 2020, the answers to many research questions on the COVID-19 pandemic have become much clearer [1]. Still, some important dilemmas remain relevant as we enter the new year 2021.

Why did almost two billion people in Asia barely feel the effects of the pandemic, while two billion people in Europe, North and South America were burdened with a large number of sick and dead people, as well as significant economic damage? Countries in Asia such as South Korea, Singapore, China, Japan, Taiwan, Vietnam, Cambodia, Laos, Bhutan, Thailand and Mongolia – the populations of which when combined together number close to two billion people – managed to get themselves through most of 2020 with ‘’textbook examples” of the application of anti-epidemic measures which discouraged the spread of the virus among their populations. They achieved this by introducing tight controls on their borders and implementing a mandatory two-week quarantine for all persons who entered their territories. Each case of infection was dealt with very seriously, and all contacts of those who become infected were monitored and isolated. The population followed the instructions of their epidemiologists, wore masks and maintained the measure of social distancing. In this way, the aforementioned Asian countries saved the lives of their fellow citizens, as well as economic activities, resulting in them maintaining a fairly ordinary everyday life [2].

How and why have these Asian countries managed to suppress the pandemic to such an extent that some even predicted economic growth in 2020, while the entire developed Western world, which is on average significantly more wealthy, has suffered severe public health and economic crisis due to the pandemic? Even in fifty to one hundred years in time, it will not be easy for historians to understand why Western countries didn’t introduce the appropriate epidemiological measures in the fight against the spread of a dangerous infectious disease. The aforementioned Asian countries have learned lessons from their previous experiences of the epidemics of SARS and MERS, and as such have prepared very well for the suppression of a new epidemic [2,3].

Western countries were hesitant and late in taking appropriate measures. This allowed the virus to spread freely within the borders of those countries. This, in turn, led to both a public health and an economic crisis. Such hesitation was often motivated by the desire to disrupt economic activities as little as possible, but also by scepticism when it comes to whether or not the population would accept very strict measures such as monitoring their contacts and isolating them. On top of that, there was also the unrealistic hope that the virus might not be so harmful in their country. However, any underestimation of the virus would prove dangerous over time. As a rule, it would lead to the prolonged duration of anti-epidemic measures, as well as even greater damage to the economy [4].

How to explain the reluctance of developed Western countries to introduce anti-epidemic measures, the opposition of part of the population to those measures, and the relative passivity and tolerance towards significantly higher numbers of infected and dead people when compared to the most successful countries in Asia? “Textbook” epidemiology implies prioritising the control of dangerous infectious diseases and the full cooperation of the population. This is because this control should be in the interest of everyone in society – taking into account the points of view of public health, economics and security. That is why, during the very first days of the pandemic of COVID-19, epidemiologists didn’t even take into account that there could be any hesitation when it came to applying strict anti-epidemic measures anywhere in the world, let alone the deliberate postponement of such measures [5,6].

After mass vaccinations took place in the second half of the 20^th^ century, the countries of the developed Western world weren’t encountering any dangerous epidemics. Therefore, today some of the leading epidemiologists of such countries believe that the sense of great danger of the spread of infection, which is still present in less developed countries today, has been “depleted” among Western populations. The less developed countries still register significant annual mortality from tuberculosis, AIDS, malaria, various tropical diseases, as well as diseases among pregnant women and childhood infections such as pneumonia, diarrhoea, meningitis and sepsis. Therefore, the poorer countries of the world still take the epidemic spread of infectious diseases extremely seriously [6].

Before the onset of this pandemic, commentators in the global media dealt with the possible influences of the phenomenon of “post-truth” and “post-capitalism” on Western civilisation [7,8]. After the pandemic, some other topics may emerge as even more important – and “post-education” and “post-reason” may be discussed in the coming months and years. A good education, critical thinking, rationality, pragmatism and common sense were once valued in Western countries. Even the members of the lowest social strata aspired to these goals for themselves and for their children. However, what happened with the waves of the pandemic in the US, such a rich and prosperous country – especially the third one – is probably very difficult to understand for any experts in the prosperous countries of Asia.

The third wave which spread across the US was significantly worse than the first two, and the death toll has exceeded 500 000 and will continue to rise. That means that nearly two Americans in every 1000 inhabitants have already died. Some European countries currently have even worse relative indicators following their second wave – eg, Belgium, the Czech Republic, Hungary, Italy, United Kingdom, and several Eastern-European countries. In addition to this tragedy, the US Center for Disease Control is recording an excess of at least an additional 100 000 deaths when compared to the averages of previous years, which have yet to be explained [9,10].

It has become obvious that decades of fairly secure and quality life, constant economic growth, great progress in personal freedoms, as well as political aspirations for the mutual openness of borders and globalisation, have led to the indecision of the leaders of at least some Western countries in the application of anti-epidemic measures. Part of the reason may be that the basic principles of anti-epidemiological measures, in their very nature, are somewhat in conflict with those historically important social achievements of the Western world.

Some of the leaders of the West may have assumed that it would be easier for their people to accept the deaths of many senior citizens and the economic downturn than for them to accept the strict and consistent implementation of nationwide anti-epidemic measures, such as those seen in Singapore, China or South Korea, which puts the interests of the whole community first, but sometimes interferes with the personal freedoms of individuals. This is an interesting 21^st^-century phenomenon that will be studied for years to come.

Based on the experiences of almost 200 countries and areas in the world today during 2020, can we talk today about a scientifically optimised approach to pandemic control in 2021? During the first few months of the pandemic, there were too many unknowns and unanswered questions for any country’s response to be considered “optimal.” In addition, the adequacy of that response depended significantly on two key criteria that seemed to contradict each other – reducing the number of victims of the pandemic and preserving economic activity. Many believed that the countries which managed to find the best trade-off between saving human lives and preserving economic activity would be the ones whose response would prove to be the most successful as time went on.

However, from month to month, it turned out that the two criteria were not as contradictory as they initially seemed to be. Simply put, the decisive and successful control of the pandemic also made it easier to preserve economic activity, while an excessively widespread contagion significantly undermined the economy, too. Understanding that without tight pandemic control there is no help for the economy has led several Western countries, encouraged by this insight, to increasingly adopt elements of the strategy of the most successful Asian countries during the pandemic’s second wave.

As such, we have seen that Norway, Finland, Denmark and Iceland, but also Australia and New Zealand decided to try to reduce the number of infected people within their borders down to a minimum. They accepted that there was now enough scientific evidence that it was better for society, as a whole, to have as few worries about the virus as possible than to constantly fight it and live in an atmosphere of uncertainty. If this virus is underestimated or ignored in any way, it soon becomes the main topic. It paralyses the society with its rapid spread. When hospitals fill up, people retreat to their homes despite the anti-epidemic measures, and this harms the economy even if those measures are relatively mild [11].

In the meantime, another extremely important change has taken place: effective and safe vaccines against the SARS-CoV-2 virus have been developed. Given the possibility of deploying these vaccines in the coming months, now each country has an additional imperative to save as many human lives as possible. At the same time, good control of the epidemic should preserve as many economic activities as possible. This is one of the most important reasons why many European Union countries opted for very strict anti-epidemic measures during November and December 2020, some even opting for lockdowns and curfews, and then continued with this policy into 2021. Quite simply, based on the growing amount of scientific evidence, they decided to reduce the problem caused by the COVID-19 pandemic as much as possible within their borders, especially in view of emerging new and mutated strains that spread much faster [12].

Therefore, the approach currently supported by science is very simple: countries that apply strict anti-epidemic measures will succeed in curbing the pandemic, and thus preserve their economy. Numerous excuses for delaying application of measures have slowly proved inconsistent. As Tomas Pueyo hinted in his many widely read communications on the pandemic, accusatory claims that “only countries inclined to totalitarianism can truly curb a pandemic” have been refuted by the examples of New Zealand, Japan and Australia. False beliefs that “only islands can truly control their borders” have been refuted by the examples of China, Thailand and Vietnam. These three countries, as well as Cuba, Mongolia and some African countries, have also worked to deny the misconception that countries „need to be very rich in order to successfully combat a pandemic”. The idea that the “Anglo-Saxon nations were too free to be subject to the strictest measures” was refuted by the examples of Australia and New Zealand. Simply put, it became reasonable to curb the pandemic in late 2020, thus protecting both the economy and waiting for vaccination to begin, and all other approaches are slowly becoming dubious [13].

Has the insight that the economy is best protected by pandemic control, and the emergence of effective and safe vaccines, resolved the conflict of opinion between scientists who have signed the so-called “John Snow” memorandum and those who supported the so-called “Great Barrington” declaration [14,15]? That conflict escalated at an earlier stage of the pandemic when there seemed to be a possibility that we would be trapped between these two bad options for too long and the population would get tired of adhering to anti-epidemic measures. And indeed, if there had been no progress in the life-saving hospital treatment and development of the vaccine, and if we were facing at least two or three more years of such captivity, this conflict would have gained in its importance. The signatories and supporters of the “Great Barrington” declaration saw a way out of such a situation in the normalisation of life, the neglect of pandemic control, but with special protections for the elderly and the most vulnerable. They were of the opinion that the epidemic would somehow have to limit itself over time and that the total damage to society, in that case, would ultimately be the least [15].

However, with the advent of vaccines, the idea of neglecting the control of the spread of infection quickly lost its importance. There is no one among the ranks of responsible epidemiologists who thought that at the end of 2020 there was any human population in the world, except perhaps in Manaus, Brazil and smaller parts of Lombardy, that had enough people immune through exposure to expect a significant slowdown in infection rates due to “collective immunity”. Even in the most severely affected countries of the world, the number of those infected was not even close to any threshold that would be needed to achieve collective immunity [16].

In addition, epidemiologists know that the percentage of people who will become infected during the free spread of an epidemic will eventually be higher than the threshold required to achieve collective immunity because of so-called “epidemiological overshooting”, ie, the excess of the number of those infected which occurs even after the achievement of collective immunity, when the free spread of the epidemic continues [17].

Furthermore, epidemiologists clearly distinguish that there are people who are exposed to the virus and the presence of the virus within them can be proven by testing, but this still does not necessarily mean either the appearance of any symptoms or the development of immunity. Therefore, the total number of people exposed to the virus who have tested positive doesn’t have to match the number of people who have actually acquired immunity to the virus.

Moreover, epidemiologists know that there is a certain “gradient” in acquired immunity to the virus: the more severe the symptoms of COVID-19, the longer the immunity will last, but the milder the clinical picture is, the shorter it will last. In some people exposed to the virus with very mild or no symptoms at all, immunity can last for a very short time. Therefore, people who have already been exposed to the virus have the susceptibility to a new infection with the same virus returned to them once again, thus again reducing the threshold of collective immunity, as they can be infected all over again [18].

Finally, the virus itself is constantly mutating, and previously acquired immunity does not always have to protect us from new strains. In principle, therefore, a new strain of virus may require a new vaccine. Therefore, it isn’t a good strategy to allow for mass infection with the virus, as this increases the likelihood of new and potentially more dangerous strains. The emergence of new, mutated strains in the UK, South Africa, Brazil and elsewhere, which are spreading much more rapidly, is very worrying news for everyone.

This is because this increased rate of spread also means an even significantly higher threshold required to reach collective immunity, ie, that 80% or 90% of people will need to be vaccinated to achieve herd immunity, instead of 70%. This threshold becomes significantly more difficult to achieve. In addition, stricter anti-epidemic measures are needed to slow down the spread of these new strains, as the ones that are now in place will no longer be enough. Furthermore, such strains will cause the emergence of new cases even more quickly, which will require hospital care.

There is also the problem of so-called “long COVID”, ie, long-lasting symptoms that linger for months in a considerable proportion of people who have survived the disease. There are also a number of as-yet-unexplained health problems that COVID-19 can cause in those who get over the infection. These are all reasons why any thinking about “collective immunity” by mass infection is not being considered in countries that rely on scientific achievements in their planning. It is an educated and reasonable act from the governments now to first protect the population from infection by decisive anti-epidemic measures and wait for the vaccine. A number of countries are already doing so.

Unless, of course, you live in one of the western countries that are beginning to experience the novel challenges of “post-education” and “post-reason”. The emergence of groups in the community that are now openly “anti-measures” resembles one of modern “anti-vaxxers”, that emerged a few decades earlier. Both of these ideas seem to have their roots in the lack of ability to recognise the reliable scientific facts and separate them from the apparent misinformation, lack of critical thinking that should have been acquired through education and the inability to make a reasonable assessment of the optimal way out of this crisis that would not be impatient or primarily self-interested.
